# The impacts of public hospital comprehensive reform policies on hospital medicine cost, revenues and healthcare expenditures 2014–2019: An analysis of 103 tertiary public hospitals in China

**DOI:** 10.3389/frhs.2023.1079370

**Published:** 2023-03-07

**Authors:** Lin Pan, Kai Xiao, Huanhuan Zhu, Li Luo

**Affiliations:** ^1^ School of Public Health, Fudan University, Shanghai, China; ^2^ Shanghai Institute of Infectious Disease and Biosecurity, Shanghai, China; ^3^ Department of Medical Administration, Shanghai Jiading District Health Commission, Shanghai, China

**Keywords:** China, the comprehensive public hospital reform policy (CPHRP), difference-in-difference (DID), the impacts, public hospitals

## Abstract

**Objective:**

To explore the impact of implementation of the comprehensive public hospital reform policy (CPHRP) on medicine costs, revenues and medical expenditures in tertiary public hospitals in China.

**Methods:**

The data of this study was collected from local administrations to obtain operational data of healthcare institutions and medicine procurement data for 103 tertiary public hospitals from 2014 to 2019. The propensity matching score method and the difference-in-difference method were used jointly to assess the impact of reform policies on tertiary public hospitals.

**Results:**

After the implementation of the policy, drug revenue in the intervention group decreased by ¥ 86.3 million (*p* = 0.076) compared to the control group; medical service revenue increased by ¥ 108.5 million (*p* < 0.001); government financial subsidies increased by ¥ 20.3 million (*p* = 0.085); the average medicine cost per outpatient and emergency visit decreased by ¥ 15.2 (*p* = 0.062); the average medicine cost per hospitalization decreased by ¥ 504 (*p* = 0.040); however, the medicine cost decreased by ¥ 38.2 million (*p* = 0.351), the average cost per visit for outpatient and emergency decreased by ¥ 0.562 (*p* = 0.966), the average cost per hospitalization decreased by ¥ 152 (*p* = 0.844), which are not significant.

**Conclusions:**

The implementation of reform policies has changed the revenue structure of public hospitals; the proportion of drug revenue decreased, while the proportion of service income increased, especially in service income and government subsidies. Meanwhile, the average medicine cost of outpatient, emergency, and inpatient visits per time were all reduced, which played a certain role in reducing the disease burden of patients.

## 1. Introduction

The high growth rate in total health expenditures has become one of the major global concerns (1, 2). In China, pharmaceutical expenditure is a major component of healthcare expenditure. Since the start of the reform and opening-up policy in 1978 until 2012, China's expenditure on pharmaceuticals as a proportion of healthcare expenditures has been well above the overall level of 20% in Organization for Economic Cooperation and Development (OECD) countries and has always exceeded 40 percent (3, 4). The reason for the above phenomenon is that since 1978, China implemented a “mark-up policy” aimed at addressing the significant insufficient government financial subsidies to public hospitals and the fact that the benefits of medical services are far below the actual costs (5, 6). The implementation of the “markup policy” has also created negative problems. For example, considering the potential benefit delivery, the actual revenue of some medicines in public hospitals may reach about 40% of the price. The reason for this phenomenon is that in order to increase revenue, public hospitals may have engaged in hidden deals with medicine suppliers, in the process of drug procurement and prescription, a practice that defeats the normal mechanism of price competition (7). In the interests of financial incentives, doctors might prescribe more drugs than were needed, with higher profit margins (8, 9). Between 1990 and 2008, the average outpatient expenditure on medicines grew even faster than the gross domestic product (GDP) over that period, rising exponentially from ¥ 10.90 to ¥ 146.50 (10).

Since the decision-making of prescription drug consumption is the key link between drug production and consumption, the medical institutions that collect doctors' prescribing rights are the key player in the drug industry chain. So the behavioral preferences of public hospitals will influence the choice of varieties and quantities of medicines, which will ultimately be reflected in the usage and structure of the cost of medicines. In terms of the market share of drug terminals, the proportion of drug costs in medical institutions in the total drug costs has maintained at a high level of 65%–70% in recent years, playing a leading role in the demand side of the drug market. Therefore, policies governing public hospitals are crucial to controlling the cost of medicines. As an important part of the efforts to deepen healthcare reform, the comprehensive public hospital reform policy (CPHRP) is dedicated to improving the level of governance in public hospitals. It focuses on lowering the price of medicine and thus providing affordable and high-quality healthcare services to residents (11). In 2010, China's Ministry of Health and five other ministries and commissions jointly issued the “Guidance on Public Hospital Reform Pilot”, selecting 16 cities as pilot areas for public hospital reform under national guidance, and the comprehensive reform of public hospitals was officially launched (12). Subsequently, in May 2014 and 2015, the Office of the Leading Group for Deepening the Reform of the Medical and Health System identified the second and third batches of pilot cities for public hospital reform respectively, and by 2017, the pilot comprehensive reform of urban public hospitals was fully rolled out (13). All public hospitals have implemented a series of reform policies with mark-up policy of medicines as a breakthrough, which include adjusting the price of medical services, increasing financial input from the government, reforming payment methods, improving the management system and operation mechanism, reforming the personnel and remuneration system and other aspects. Some of these policies have a significant impact on the cost of pharmaceuticals.

The comprehensive reform of public hospitals to abolish drug mark-up policy and implement zero-rate sales of medicines as a breakthrough refers to changing the funding sources for public hospitals from three channels of service charges, drug mark-up income and government subsidies to two channels of service charges and government subsidies (7).

The adjustment of medical service prices includes the reduction of the cost of medicines and medical consumables, the abolition of the drug mark-up policy, the reduction of the price of large medical equipment examination, and the reasonable adjustment and upgrading of the price of medical services that reflect the value of the technical labor of medical personnel, especially the price of services such as diagnosis, treatment, surgery, nursing, etc (14). Increasing government investment is to guarantee the funding required for public services undertaken by public hospitals. The main measures are to implement investments in public hospital capital construction and equipment acquisition, development of key disciplines, talent training, retiree costs in line with national regulations and policy loss subsidies, and to provide special subsidies for public health tasks undertaken by public hospitals (15, 16). The implementation of the reformed payment method is based on a total budget, which adapts to a composite payment method with different population groups, different diseases and characteristics of medical services. This payment method gradually reduces the payment by items and covers all medical institutions and medical services. The composite payment method can give full play to the incentive and restraining effect of basic medical insurance and control the unreasonable increase of medical expenses. It can promote the initiative of medical institutions and medical personnel to control costs and expenses and improve the efficiency of the use of medical resources and funds, thus reducing the burden of medical costs on participants at source (14). Improving the management of medical institutions mainly includes the establishment of an assessment and evaluation mechanism oriented to public welfare. The reform of the personnel remuneration system includes the establishment of a remuneration system that is in line with the characteristics of the medical and healthcare sector and that reflects the value of the technical services of the medical staff and sets a reasonable level of income for them (17).

Previous studies assessing the effectiveness of CPHRP have focused on county-level public hospitals or a particular geographical area. In addition, most of these studies have centered on healthcare expenditures, the cost of drugs and health service utilization (16, 18–21). Studies in China have found that public hospital reforms reduced drug costs, but it has not been effective in curbing the increase in healthcare expenditure. A study from Zhejiang Province of China (21), concentrating on the effect of CPHRP on health expenditure and health service utilization in county-level hospitals, found changes in the composition of hospital revenues, a decrease in average per outpatient and inpatient drug costs, but an increase in average per outpatient and inpatient costs after the reform was initiated. In addition, a study of the effect of CPHRP on hospitalization costs in Hubei found that the reform reduced inpatient spending on medications but did not lead to lower personal health expenditure (18). A study of county-level public hospitals at the national level also found that public hospital reform reduced drug spending, but the effect of reducing total health expenditure remained limited (22). A study quantified the overall and dynamic impact of the zero-plus-price drug policy on the proportion of revenue from drugs, medical services and government subsidies in China's tertiary public hospitals, and it found that implementation of the policy adjusted the revenue structure of public hospitals, with the share of the revenue from drugs continuing to fall, the share of the revenue from medical services continuing to rise, and the share of the revenue from government subsidies changing little (7).

Studies addressing the impact of policy factors such as revenue and healthcare expenditure in public hospitals mainly focused on primary care facilities and county-level hospitals, with only a few studies examining tertiary public hospitals (23–25). There are differences in the volume and level of services provided by urban public hospitals and county hospitals, so it is difficult to extrapolate the findings of studies on county hospitals to urban tertiary public hospitals (26). The available evidence on the impact of CPHRP on public hospitals in China also focuses on a sample of hospitals in a particular area, with even fewer studies at the national level on urban tertiary public hospitals (22). Research findings that are limited to a particular region may also be influenced by factors such as the level of economic development of the region, making it difficult for the findings to reflect the impact of the policy at a national level.

The samples of this study are tertiary public hospitals at the national level. The researcher's objective was to explore the impact of public hospital reform policies on healthcare expenditures, drug costs and revenues in tertiary public hospitals. We analyzed medical institution operation data and medicine procurement data from 103 tertiary public hospitals across China from 2014 to 2019. We quantify the changes in indicators such as the cost of medicines, revenues and average costs per patient visit of these public tertiary hospitals nationwide before and after the implementation of the CPHRP. This study aims to determine whether the implementation of the CPHRP can adjust the revenue structure of medical institutions and achieve policy objectives such as reducing healthcare expenditure.

## 2. Materials and methods

### 2.1. Research design

This study assesses the impact of CPHRP on medicine costs, medicine and other revenues, and health care expenditures of sample hospitals through the difference-in-difference (DID) method, based on panel data from 103 public tertiary hospitals in China from 2014 to 2019. Based on the implementation of the CPHRP in each province, the sample hospitals that initiated comprehensive public hospital reform in 2017 were selected as the intervention group and those that initiated reform before January 1, 2016, as the control group. On this basis, the effect of the reform policy was evaluated by the DID method. The DID method is an effective tool to evaluate the effects of policy implementation and it is used to quantitatively assess the effectiveness of public policy or program implementation. This approach controls for the effects of certain factors other than the intervention policy, effectively separating the true results of the policy impact. It divides the study population into an intervention group with a changing policy environment and a control group with a generally stable policy, then calculates the amount of change in the same indicator before and after the implementation of the policy for the intervention and control groups respectively, and finally calculates the difference between the above two changes, which is the difference between the two groups after excluding the influence of other factors, i.e., the net effect of the reform policy on this indicator (7, 27).

### 2.2. Sample size, data source and study indicators

The target hospitals for this study were identified using a quota sampling method. We selected three provinces in each of the Eastern, Central and Western regions of China, for a total of nine provinces as the sample areas for this study. We chose sample areas taking into account the different levels of economic and social development in the region: the number of sample hospitals in the Eastern regions, Central regions and Western regions was 55, 49 and 64 respectively. The sample hospitals were selected based on the following considerations: (1) The type of medical institution is a tertiary public hospital; (2) To ensure the stability of the selected institutions, medical institutions with a change rate of 15% or less in the number of beds and staffing between 2014 and 2019, without major renovation and expansion projects, and not involving major changes such as merging and splitting of institutions, were selected; (3) Medical institutions with continuous, complete and standardized procurement data in the provincial drug procurement platform and a stable net procurement rate of over 90% were selected.

We first initially selected 91 sample hospitals that initiated public hospital reform policy before 2016 as the control group and 77 sample hospitals that initiated public hospital reform policy in 2017 as the intervention group. To reduce the effects of differences in service capacity levels between sample hospitals and other confounding variables on the results, we applied the propensity score matching method (PSM) for matching and finally identified 51 sample hospitals in the intervention group and 52 sample hospitals in the control group, for a total of 103 tertiary public hospitals, as the sample hospitals in this study.

The data for this study are operational data of healthcare institutions from 2014 to 2019, which were collected from local administrations and sample hospitals. Data on the operation of medical institutions include the level of institution, number of beds, the total number of professional and technical staff, revenues (revenue from medicines, revenue from health services, revenue from government financial subsidy), cost of patient visits (average outpatient and emergency cost per visit, average outpatient and emergency medicine cost per visit, the average cost per hospitalization, average medicine cost per hospitalization, etc. The cost of medicines is the costs of medicines consumed by healthcare providers in the course of their healthcare services and ancillary activities.

The medical revenue of a medical institution is the sum of outpatient revenue and inpatient revenue. Medicine revenue is the sum of medicine revenue from outpatient and emergency and medicine revenue from inpatient services. Medical service revenue includes consultation revenue, pharmacy service revenue, surgery revenue, nursing revenue, etc., but it does not include revenue from medicines, health consumables, examinations and tests, etc.

1. Average cost per visit for outpatient and emergency = revenue from outpatient and emergency/number of outpatient or emergency visits;
2. Average medicine cost per visit for outpatient and emergency = medicine revenue from outpatient and emergency/number of outpatient or emergency visits;
3. Average cost per visit for hospitalization = revenue from hospitalization/number of hospitalization visits;
4. Average medicine cost per visit for hospitalization = medicine revenue from hospitalization visits/number of hospitalization visits.

### 2.3. Statistical analysis

#### 2.3.1. Descriptive statistical analysis

In this research, we use descriptive statistical analysis to describe the basic characteristics of sample hospitals, medicine costs and revenues of the sample hospitals, and the average cost or medicine cost per outpatient and emergency or hospitalization.

#### 2.3.2. Propensity score matching

To reduce the effect of differences in service capacity levels between the sample providers and other confounding variables on the results, the PSM method was applied by matching individual characteristics such as service capacity and efficiency between the two groups of hospitals. We first selected medical institutions of the same level and calculated propensity scores based on indicators of medical institutions' capacity and efficiency. In order to form pairs, we next searched for sample objects in the control group which was closest to the intervention group sample score using a forward or backward approach by nearest neighbor matching. Finally, we identified sample hospitals for the post-matching intervention and control groups.

#### 2.3.3. The DID method

The DID method was used to analyze the effects of the CPHRP, the formula is as follows:

$$\begin{array}{rl} d_{ID}= & (Y_{Intervention\;group,t_{i}}-Y_{Intervention\;group,t_{0}})\\ & \;-(Y_{Control\;group,t_{i}}-Y_{Control\;group,t_{0}}) \end{array}$$

$d_{ID}$ represents the double difference value of the policy on indicators such as those related to the sample hospitals in year i, which in this study are the double difference values of medicine revenue, medicine cost, medical service revenue, government financial subsidy revenue, the average cost per outpatient and emergency, average medicine cost per outpatient and emergency, average cost per hospitalization, and average medicine cost per hospitalization; $Y_{Intervention\;group},t_{0}$ denotes the level of the relevant indicator for the intervention group in the baseline year; $Y_{Intervention\;group,t_{i}}$ denotes the level of the relevant indicator for the intervention group in year; $Y_{Control\;group,t_{0}}$ indicates the level of the relevant index in the control group in the baseline year; $Y_{Control\;group},t_{i})$ denotes the level of the relevant indicator for the control group in the year *i*.

There are many factors influencing the medication preference of medical institutions, the traditional DID method does not take into account the characteristics of individual differences of medical institutions, and the validity of the analysis results is affected by the absence of relevant variables. The PSM method is effective in reducing confounding bias in observational studies and gets similar results to randomized controlled studies throughout the study design phase. In this study, to reduce the effect of confounding factors in the intervention group and control group, a Logistic regression model was used to match the policy and control groups, the formula is as follows:

$$Logit[Pr(Y_{i}=1)]=\beta_{0}+\sum \beta_{i}\;X_{i}+\eta_{i}$$

$Y_{i}$ indicates whether it is in the intervention group, 1 is the intervention group, and 0 is the control group; $Pr(Y_{i}=1)$ indicates the probability that the sample hospital is included in the study sample; $X_{i}$ refers to the control variables of medical institution characteristics, including the number of health technicians, the average length of stay, the number of beds, the bed utilization rate, and the proportion of tertiary and quaternary surgeries; $\beta_{i}$ refers to the regression coefficients of the corresponding control variables; $\beta_{0}$ is a constant term; and $\eta_{i}$ is a random disturbance term.

## 3. Results

### 3.1. Characteristics of sample hospital

Based on the implementation of the CPHRP across China, 91 sample hospitals that initiated public hospital reform before January 1, 2016, were initially selected as the control group, and 77 sample hospitals that initiated reform in 2017 were selected as the intervention group.

In this study, PSM was used to reduce the effect of differences in service capacity levels between the sample hospitals and confounding variables on the study results. There were 51 sample hospitals in the intervention group and 52 sample hospitals in the control group after matching. The basic characteristics of the sample hospitals and their comparison are shown in Table 1.

**Table 1:** Characteristics of the sample medical institutions before and after PSM.

	Before PSM			After PSM		
	Control group	Intervention group	*p*-value	Control group	Intervention group	*p*-value
Number of sample hospitals	91	77		52	51	
Number of Beds	1,370	1,456	0.037	1,386	1,350	0.424
Number of health technicians	1,431	1,564	0.018	1,435	1,487	0.407
Average hospital stay (day)	9.237	9.074	0.014	9.121	9.275	0.142
Efficiency of bed utilization (%)	96.89	95.61	0.008	96.12	95.71	0.443
Number of inpatient surgeries (million)	1.870	2.145	<0.001	1.979	2.014	0.898
Ratio of tertiary and quaternary surgeries (%)	46.54	47.45	0.078	47.14	47.22	0.643

Calculated using data from sample hospitals from 2014 to 2019.

As shown in Table 1, several variables were significantly different between the two groups of hospitals before matching and the differences in the indicators were not statistically significant after matching. These data suggest that PSM is effective.

In general, the distribution of propensity scores of the two groups of hospitals is relatively similar, which meets the requirement of feature score matching. So the DID method can be used to further analyze and evaluate the effect of the comprehensive public hospital reform policy.

### 3.2. Changes in hospital medicine costs, revenues and patient medical costs during the implementation of the reform policy

**Figure 1:**
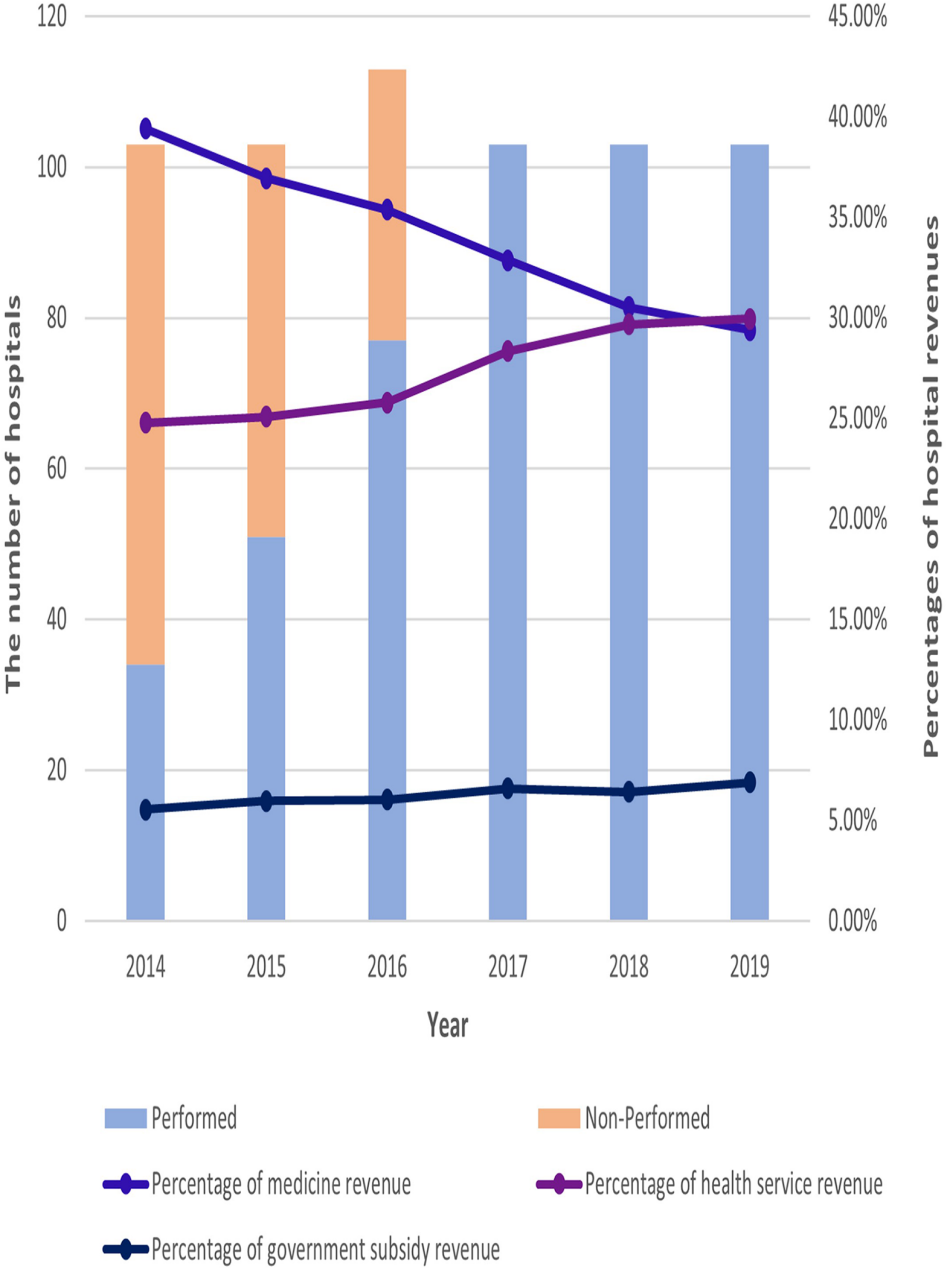
Gradual implementation of policies and changes in revenue share for 103 hospitals from 2014 to 2019.

During the implementation of the public hospital reform policy from 2014 to 2019, the proportion of medicine revenue in the total revenue of public hospitals showed a gradual decline, and the proportion of medical service revenue and government subsidies revenue in total revenue showed a gradual increase. For more details, see Figure 1.

**Figure 2:**
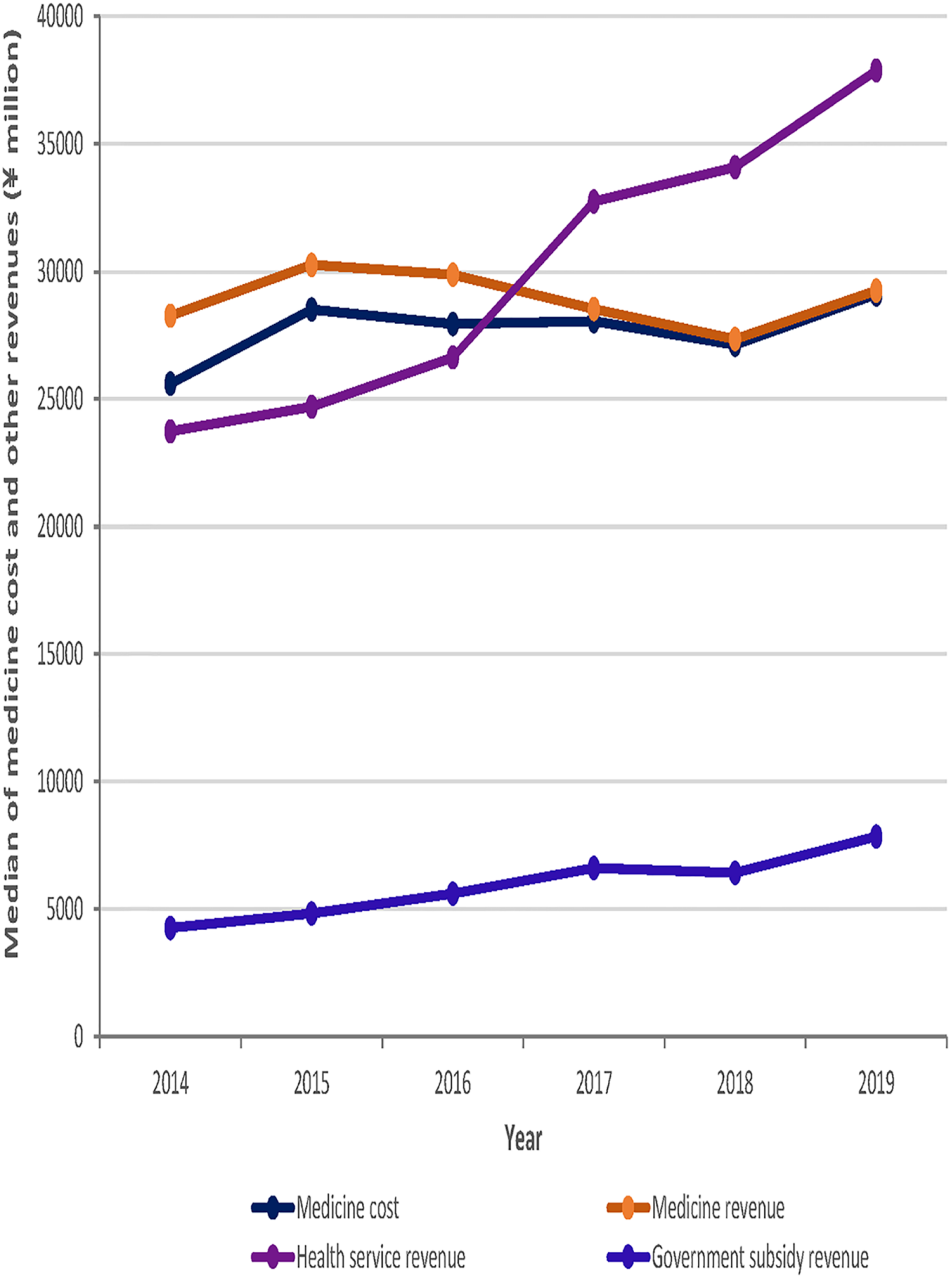
Trends in median medicine costs and revenues for the 103 sample hospitals from 2014 to 2019.

As shown in Figure 2, medicine costs in the study sample hospitals showed an upward trend between 2014 and 2019, with the median drug cost rising by ¥3.507 million on average per hospital over the six-year period. Medicine revenues for healthcare organizations showed an overall downward trend through 2018 and a slight upward trend from 2018 to 2019. Government subsidy revenue showed an upward trend, with the median government subsidy rising by a total of ¥ 3.581 million on average per hospital from 2014 to 2019. Medical service revenue showed an upward trend over the six-year period, with median medical service revenue rising by a total of ¥ 14.145 million on average per hospital. Revenue from medical services rose the fastest in the year 2016–2017, in which all sample institutions were about to implement the public hospital reform, rising by ¥ 6.112 million on average per hospital during the one-year period.

**Figure 3:**
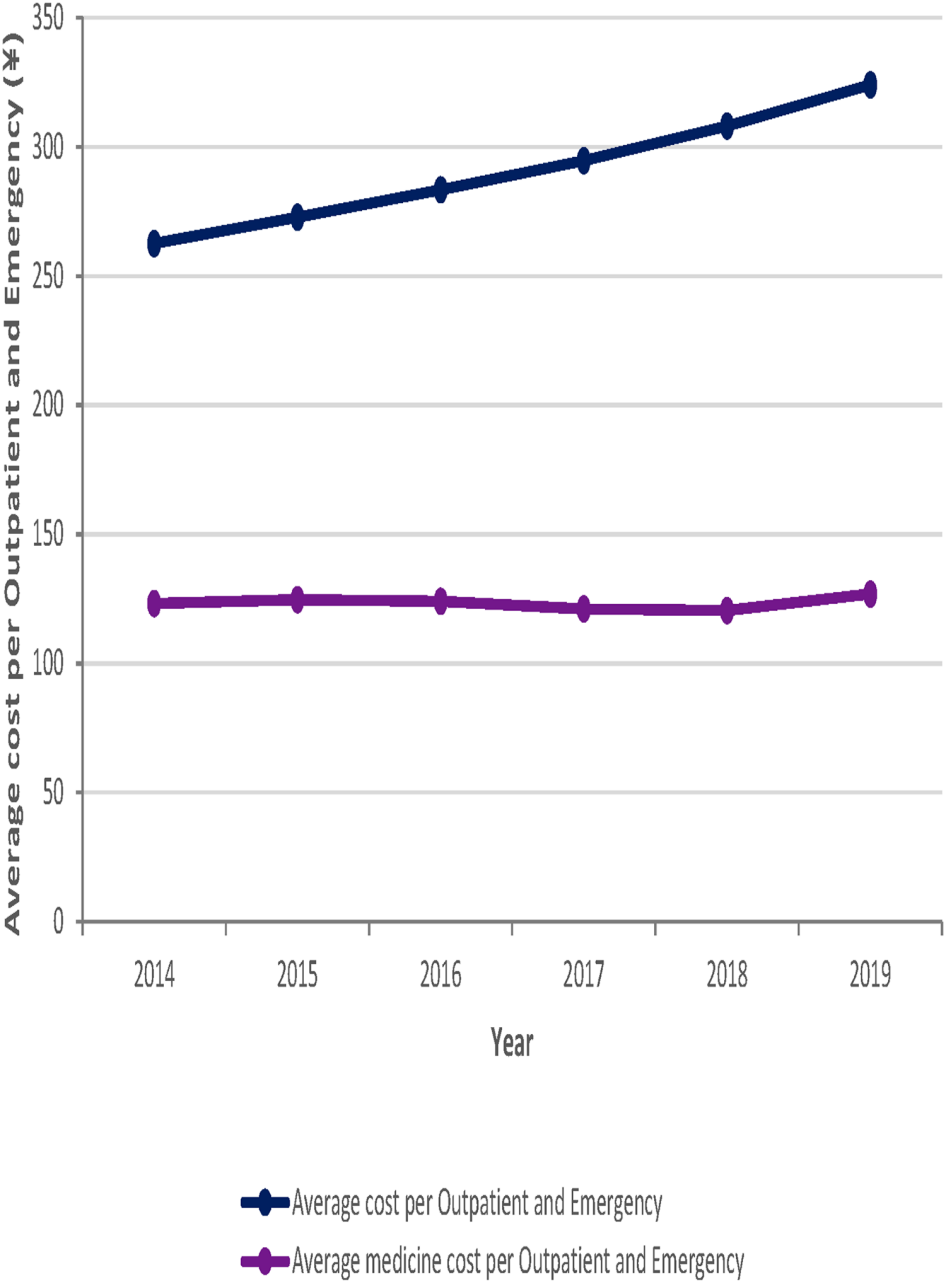
Trends in average cost or medicine cost per Outpatient and Emergency for the 103 sample hospitals from 2014 to 2019.

**Figure 4:**
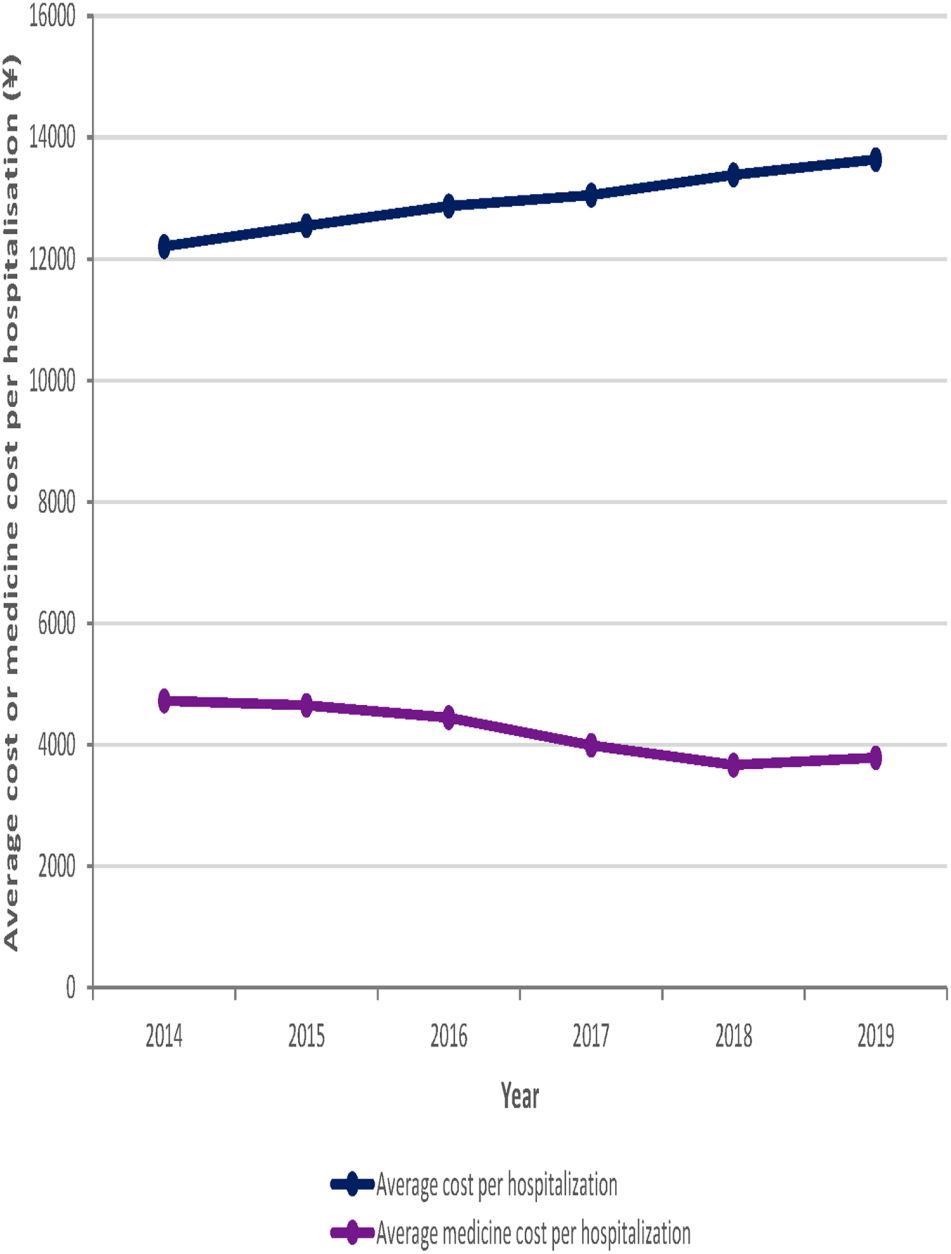
Trends in average cost or medicine cost per hospitalization for the 103 sample hospitals from 2014 to 2019.

During the six-year period from 2014 to 2019, the average per outpatient and emergency cost and the average per inpatient cost of the study sample institutions showed an upward trend, rising by ¥61.4 and ¥1430.2, respectively. However, the average medicine cost per outpatient and emergency visit showed a trend of a smaller range of fluctuating changes. In addition, the average medicine cost per hospitalization showed an overall downward trend, decreasing by a total of ¥ 938.2 from 2014 to 2019. See Figures 3, 4 for more details.

### 3.3. The impact of the CPHRP on hospital medical costs, revenues, and patient medical costs

As shown in Table 2, compared with the control group, hospitals in the intervention group before and after the implementation of the CPHRP had a decrease in medicine revenue of ¥ 86.3 million, a decrease in the costs of medicine of ¥ 38.2 million, an increase in medical service revenue of ¥ 108.5 million, and an increase in government subsidy revenue of ¥ 20.3 million.

**Table 2:** The changes in medicine costs and revenues in the sample hospitals.

	Intervention group	Control group	Differences between groups
**Medicine cost**
Pre-implementation	353.8	369.9	−16.1
After implementation	367.9	422.2	−54.3
Time Difference	14.1	52.3	
Difference in Difference			−38.2
**Medicine revenue**
Pre-implementation	404.5	383.7	20.8
After implementation	372.4	437.9	−65.5
Time Difference	−32.1	54.2	
Difference in Difference			−86.3*
**Health service revenue**
Pre-implementation	236.6	312.1	−75.5
After implementation	404.5	371.5	33.0
Time Difference	167.9	059.4	
Difference in Difference			108.5
**Government subsidy revenue**
Pre-implementation	58.1	64.3	−6.2
After implementation	95.8	81.8	14.1
Time Difference	37.7	1.75	
Difference in Difference			20.3*

* *p* < 0.1.

The multiplicative differences between medicine revenues (*p* = 0.076) and medicine costs (*p* = 0.351) were negative and the multiplicative differences between medical service revenues (*p* < 0.001) and government subsidy revenues (*p* = 0.085) were positive. The above results indicate a significant decrease in medicine revenues and a significant increase in medical services revenues and government revenues for medical institutions after the implementation of the comprehensive public hospital reform policy, while the CPHRP did not have a significant effect on the change in the costs of medicine.

As shown in Table 3, the multiplicative differences between the two groups of hospitals before and after the implementation of the reform policy were negative for average cost per visit for outpatient and emergency care (*p* = 0.966), average medicine cost per visit for outpatient and emergency care (*p* = 0.062), the average cost per visit for hospitalization (*p* = 0.844) and average medicine cost per visit for hospitalization (*p* = 0.044).

**Table 3:** The changes in medicine costs per visit for sample hospitals.

	Intervention group	Control group	Differences between groups
**Average cost per Outpatient and Emergency**
Pre-implementation	263.603	282.353	−18.750
After implementation	299.282	318.595	−19.313
Time Difference	35.679	36.242	
Difference in Difference			−0.562
**Average medicine cost per Outpatient and Emergency**
Pre-implementation	123.814	124.118	−0.304
After implementation	115.167	130.699	−15.533
Time Difference	−8.647	6.581	
Difference in Difference			−15.229*
**Average cost per hospitalization**
Pre-implementation	12,023	13,074	−1,051
After implementation	12,764	13,966	−1,202
Time Difference	741	892	
Difference in Difference			−152
**Average medicine cost per hospitalization**
Pre-implementation	4,532.3	4,684.7	−1,524
After implementation	3,470.0	4,126.7	−656.7
Time Difference	−1,062.7	−558.0	
Difference in Difference			−504.3*

* *p* < 0.1.

The average cost per visit for outpatient and emergency care decreased by ¥ 0.562, the average medicines cost per visit for outpatient and emergency care decreased by ¥ 15.229, the average cost per hospitalization decreased by ¥ 162, and the average medicine cost per hospitalization decreased by ¥ 504.3. The results of the above data analysis show that the comprehensive reform policies have effectively reduced patients' outpatient and inpatient medicine costs, and although the average cost per outpatient and the average cost per inpatient at the sample hospitals still increased, the growth rate was slower than that of the control group.

## 4. Discussions

Public hospital comprehensive reform policy is an important part of China's medical and health system reform because public hospitals are the most influential providers of healthcare services in China (28). Many studies have evaluated the effects of public hospital reform policy. A study conducted at the national level in China showed that the public hospital medicine and medical service price reform policies were significantly associated with reductions in average per outpatient medicine costs and average per inpatient medicine costs, and this means that the policy has initially achieved the goal of medicine expenditures being effectively reduced (26). A study conducted in Sanming City in Fujian Province showed that the public hospital reform policy significantly reduced the cost of care and did not affect the clinical quality and productivity of the hospital, due to a systemic shift in the public hospital, i.e., alignment of the hospital's governance structure, payment system, and the method of payment of physician remuneration, which was critical to improving its performance. A retrospective study conducted in Zhejiang Province showed that public hospital reform reduced the profitability of prescription medicines through the abolition of the “medicine mark-up policy” while compensating for the cost of services by raising the price of medical services. Although outpatient and inpatient medical service expenditures increased after the implementation of the reform policy, the average cost of medication per outpatient visit and the average cost of medication per hospitalization decreased (21). A study conducted in Guangxi Province to evaluate the effect of the CPHRP found that public hospital reform while controlling medical costs to some extent and improving inpatient satisfaction, did not improve the efficiency of hospital operations and had a negative impact on clinical quality (20).

Our study will provide evidence of the effectiveness of implementing the CPHRP in tertiary public hospitals in China. In this study, the PSM method and the DID method were used to count the indicators including the costs of medicine, medicine revenues, medical service revenues, government subsidy financial revenues, the average cost of medication per outpatient and emergency, the medicine cost of average per hospitalization before and after the implementation of the CPHRP in the intervention and control groups, respectively, to analyze whether the policy from 2014 to 2019 adjusted the revenue structure of tertiary public hospitals and reduced the expenditures of medical care for residents. In general, various policy factors played an important role in controlling the growth of the costs of medicine in healthcare institutions. Compared with the control group, the sample medical institutions that implemented the reform in 2017 had a significant decrease in medicine revenue, average cost of medicines per outpatient visit and the average cost of medicines per hospitalization, as well as a growth in medical service revenue and government subsidy revenue. China's public hospitals have historically been financed by medicine revenues, medical service revenues and government subsidy revenues, they are the main sources of income for public hospitals (29). The proportion of these three main components of income in public hospitals has varied over time. During the planned economy, public hospitals received most of their revenue from government subsidies, while the government regulated the prices of medical services and medicines so that residents could meet their own medical service needs at a lower cost. Since the 1980 s, as the population's demand for health services expanded and financial subsidies from the government are limited, public hospitals became responsible for their own balance sheets, they were given a certain amount of autonomy, they are allowed to get a certain percentage of profits from drug mark-ups, and drug revenues became its main source of income (30). In order to control the unreasonable increase in healthcare costs, China launched a new round of reform of its medical and health system in 2009 (31). As an important part of this reform, the implementation of the CPHRP has focused on the abolition of the drug-plus policy and the implementation of zero-rate sales of drugs, but it is worth studying whether the CPHRP has optimized the revenue structure of public tertiary hospitals and reduced the burden of medical care for residents (7). Our research found that the implementation of the CPHRP has changed the funding structure of public hospitals, it led to a decline in medicine revenues and an increase in government subsidies and medical service revenues i.e., the CPHRP had a positive impact. After the implementation of the CPHRP, both medicine revenues and the proportion of medicine revenue in total revenue showed a decreasing trend. The proportion of medicine revenue in total revenue decreased by 10.01% from 2014 to 2019 after the implementation of the reform policy. Compared to the pre-reform period, medicine revenues decreased by ¥ 8.63 million. The abolition of the medicine markup in the public hospital reform policy has achieved some effect in controlling medicine expenditures, which is consistent with previous studies (16, 32). As a breakthrough in the CPHRP, the zero markup drugs policy eliminates the profit margin of medicine sales in public hospitals, and the policy can reduce the incentive for doctors to prescribe medicines freely, thus changing the profit-making behavior of public hospitals (33).

Consistent with the findings of previous studies, our study also found that government financial subsidy revenues of public hospitals increased after the implementation of the reform policy (32, 33). The government's fiscal revenue increased by ¥ 2.03 million after the implementation of the reform policy in the intervention group. This indicates that the government, to some extent, undertook to secure the funds needed for the operation of public hospitals by providing special subsidies for the development of key disciplines and talent cultivation in public hospitals.

This study found that the CPHRP increased medical service revenues in the sample hospitals, which is consistent with the results of previous studies (7). The increase in medical service revenues may be related to the measure of changing the price of medical services in the comprehensive reform policy of public hospitals, which led to an increase in the value of medical staff's labor. In January 2017, China's National Health and Wellness Commission and other departments jointly issued the “Guidance on Conducting Pilot Work on Reforming the Remuneration System of Public Hospitals”. This document requires that Shanghai and other pilot provinces choose three cities for a period of one year each to carry out pilot reform of remuneration distribution in public hospitals. It proposes to improve the internal distribution system and distribution mechanism of public hospitals to reasonably reflect the value of medical staff's technical labor (34). Some studies show that as of 2019, only 22.58% of China's 31 provinces have completed the alignment of the old and new norms for medical service items nationwide, and the failure to implement unified medical service item norms at the national level hindered the implementation of policies to adjust medical service prices (35). Based on the above analysis, we need to consider other factors that increase revenue from medical services. The elimination of the medicine mark-up policy may cause a change in the behavior of medical services. The questions of what medical behavior changes will occur, whether such changes will affect the hospital's revenue structure, and whether this finding can be applied to other regions still need to be further investigated (7).

Consistent with the findings of previous studies, the public hospital comprehensive reform policy reduced the average medicine cost per outpatient and emergency visit and the average medicine cost per hospitalization (21, 26). The reason for this is that the public hospital reform policy of eliminating medicine mark-up policy reduced the profitability of prescription medicines (36). Our findings show that the public hospital reform policy, which aims to eliminate the use of medicines to support medical institutions, significantly reduced medicine expenditures and had some effect. China's public hospital comprehensive reform policy can reduce the financial burden on patients and increase their access to health services (37). In this study, the reform policy reduced patients' medicine costs in tertiary public hospitals. Public hospitals are the primary providers of health care services in China, providing almost 90% of all outpatient and inpatient services (21). Because of the higher level of service in tertiary public hospitals, residents are more likely to visit such providers, making the volume of services in tertiary public hospitals much higher than in county public hospitals. Therefore, studying the impact of comprehensive public hospital reform policies on reducing healthcare expenditures in tertiary public hospitals is even greater (38, 39).The following limitations exist in this study. First of all, our research was conducted on tertiary public hospitals and we can't access the data for patients for different groups, so we are unable to give a proper description of patient profiles between the different groups. Secondly, our study sample hospitals are only tertiary public hospitals and do not include primary care institutions, so we can't analyze the impact of comprehensive public hospital reform policies on different levels of medical institutions. In addition to, for public hospital revenues, this study focuses on medicine revenues, medical service revenues, and government subsidy revenues, which are not the sum of public hospital revenues, and the income of public hospitals such as scientific, educational, medical examination, medical device and other revenues are ignored. Besides, this study did not explore some reasons for the change in the income structure of hospitals i.e., the growth in medical devices and examinations, which needs to be further explored in the subsequent study. Finally, due to the large number of sample regions in this study and the differences in the level of health insurance funding, etc., among hospitals in each region, it is difficult to explore the proportion of the increased revenue from medical services paid by patients and health insurance in this study.

## 5. Conclusions

This study analyzed the effects of implementing the comprehensive public hospital reform policy in 103 tertiary public hospitals at the national level in China. The results of the study found that the implementation of the policy changed the revenue structure of public hospitals and reduced the burden of the costs of medicine on patients. The proportion of medical income in public hospitals decreased, but the proportion of medical service revenue and government subsidy revenue increased. The implementation of the comprehensive public hospital reform policy reduced the average medicine cost per outpatient and emergency visit and the average medicine cost per hospitalization, and to some extent, the policy contributed to the reduction of patient's disease burden. However, due to limitations in the availability of data, we did not analyze indicators such as access burden for different patient profiles, so in future we will focus on analyzing access burden before and after the implementation of the public hospital reform for different patients, if relevant data are available.

## Data Availability

The datasets presented in this article are not readily available because they are private medical institution data. Requests to access the datasets should be directed to Kai Xiao, xiaokaidou@163.com.

## References

[1] Parkin D. International comparisons of health expenditure. J Public Health Med. (1993) 15(1):114–5. 10.1093/oxfordjournals.pubmed.a042801 8471295

[2] Fan VY Savedoff WD. The health financing transition: a conceptual framework and empirical evidence. Soc Sci Med. (2014) 105:112–21. 10.1016/j.socscimed.2014.01.014 24524906

[3] Mossialos E Ge Y Hu J Wang L. Pharmaceutical policy in China: Challenges and opportunities for the reform. Beijing: China Development Publishing House (2017).

[4] China NHCotPsRo. Health statistics yearbook 2013. Beijing: Peking Union Medical University Publishing House (2013).

[5] China NHCotPsRo. *Health statistics yearbook 2009*. Beijing: Peking Union Medical College Press (2009).

[6] China NHCotPsRo. *Health statistics yearbook 2010*. Beijing: Peking Union Medical College Press (2010).

[7] Cheng H Zhang Y Sun J Liu Y. Impact of zero-mark-up medicines policy on hospital revenue structure: a panel data analysis of 136 public tertiary hospitals in China, 2012–2020. BMJ global Health. (2021) 6(11):e007089. 10.1136/bmjgh-2021-007089 PMC8562510 34725041

[8] Currie J Lin W Meng J. Addressing antibiotic abuse in China: an experimental audit study. J Dev Econ. (2014) 110:39–51. 10.1016/j.jdeveco.2014.05.006 26949284 PMC4776334

[9] Li Y Xu J Wang F Wang B Liu L Hou W Overprescribing in China, driven by financial incentives, results in very high use of antibiotics, injections, and corticosteroids. Health Affairs. (2012) 31(5):1075–82. 10.1377/hlthaff.2010.0965 22566449

[10] Mao W Chen W. The zero mark-up policy for essential medicines at primary level facilities. WHO. (2015). Available from: https://www.who.int/publications/i/item/WHO-HIS-HGF-CaseStudy-15.2

[11] Nong S Yao NA. Reasons behind stymied public hospital governance reform in China. PloS One. (2019) 14(9):e0222204. 10.1371/journal.pone.0222204 31498814 PMC6733505

[12] China MoPHoCSCOoPSRSDaRCMoFotPsRo. Ministry of human resources and social security opinions on the pilot of public hospital reform (2010). Available at: http://www.gov.cn/gzdt/2010-02/24/content_1540062.htm (Cited June 1, 2022).

[13] China CPsGotPsRo. Notice on the full implementation of the comprehensive reform of public hospitals (2017). Available at: http://www.gov.cn/xinwen/2017-04/29/content_5189918.htm#1 (Cited June 1, 2022).

[14] Huang J Dai T. Public hospital reforms in China: the perspective of hospital directors. BMC Health Serv Res. (2019) 19(1):142. 10.1186/s12913-019-3954-z 30819157 PMC6393992

[15] Meng Q Cheng G Silver L Sun X Rehnberg C Tomson G. The impact of China’s retail drug price control policy on hospital expenditures: a case study in two shandong hospitals. Health Policy Plan. (2005) 20(3):185–96. 10.1093/heapol/czi018 15840634

[16] Zhou Z Su Y Campbell B Zhou Z Gao J Yu Q The financial impact of the “zero-markup policy for essential Drugs” on patients in county hospitals in western rural China. PloS One. (2015) 10(3):e0121630. 10.1371/journal.pone.0121630. 10.1371/journal.pone.0121630 25790443 PMC4366182

[17] Fu H Li L Li M Yang C Hsiao W. An evaluation of systemic reforms of public hospitals: the sanming model in China. Health Policy Plan. (2017) 32(8):1135–45. 10.1093/heapol/czx058 28531288

[18] Zhang Y Ma Q Chen Y Gao H. Effects of public hospital reform on inpatient expenditures in rural China. Health Econ. (2017) 26(4):421–30. 10.1002/hec.3320 26842555

[19] Tang Y Liu C Liu J Zhang X Zuo K. Effects of county public hospital reform on procurement costs and volume of antibiotics: a quasi-natural experiment in Hubei province, China. Pharmacoeconomics. (2018) 36(8):995–1004. 10.1007/s40273-018-0654-1 29671132 PMC6021466

[20] Jiang S Wu WM Fang P. Evaluating the effectiveness of public hospital reform from the perspective of efficiency and quality in guangxi, China. SpringerPlus. (2016) 5(1):1922. 10.1186/s40064-016-3598-y 27867828 PMC5097061

[21] Zhang H Hu H Wu C Yu H Dong H. Impact of China’s public hospital reform on healthcare expenditures and utilization: a case study in Zj province. PloS One. (2015) 10(11):e0143130. 10.1371/journal.pone.0143130. 26588244 PMC4654516

[22] Fu H Li L Yip W. Intended and unintended impacts of price changes for drugs and medical services: evidence from China. Soc Sci Med. (2018) 211:114–22. 10.1016/j.socscimed.2018.06.007 29935401

[23] Tian W Yuan J Yang D Zhang L. Descriptive analysis on the impacts of universal zero-markup drug policy on a Chinese urban tertiary hospital. PloS One. (2016) 11(9):e0162795. 10.1371/journal.pone.0162795. 27627811 PMC5023112

[24] He Y Dou G Huang Q Zhang X Ye Y Qian M Does the leading pharmaceutical reform in China really solve the issue of overly expensive healthcare services? evidence from an empirical study. PloS One. (2018) 13(1):e0190320. 10.1371/journal.pone.0190320. 29338038 PMC5770029

[25] Mao W Huang Y Chen W. An analysis on rational use and affordability of medicine after the implementation of national essential medicines policy and zero mark-up policy in Hangzhou, China. PloS One. (2019) 14(3):e0213638. 10.1371/journal.pone.0213638. 30870490 PMC6417690

[26] Zhang X Lai H Zhang L He J Fu B Jin C. The impacts and unintended consequences of the nationwide pricing reform for drugs and medical services in the urban public hospitals in China. BMC Health Serv Res. (2020) 20(1):1058. 10.1186/s12913-020-05849-4 33225941 PMC7682084

[27] Wooldridge JM. Introductory econometrics: a modern approach. 5th ed. Mason, United States: South-Western Cengage Learning (2003).

[28] Wang Y Zhu Y Liu X Xu X Fang W Li X. The effects of county public hospital reform on the consumption and costs of antibiotics: evidence from a quasinatural experiment in Jiangsu, China. Biomed Res Int. (2020) 2020:9262170. 10.1155/2020/9262170. 33145360 PMC7599416

[29] Xu J Jian W Zhu K Kwon S Fang H. Reforming public hospital financing in China: progress and challenges. Br Med J. (2019) 365:l4015. 10.1136/bmj.l4015 31227512 PMC6598707

[30] Duckett J. The Chinese state’s retreat from health: policy and the politics of retrenchment. London: Routledge (2010).

[31] Shu Z Liu Y Li M Li J. The effects of health system reform on medical services utilization and expenditures in China in 2004–2015. Int Health. (2021) 13(6):640–7. 10.1093/inthealth/ihab041 34263307 PMC8643455

[32] Jiang X He P Zhu D Shi X Meng Q. Different impacts of the zero-markup drug policy on county general and traditional Chinese medicine hospitals: evidence from Shandong Province, China. Int J Equity Health. (2020) 19(1):219. 10.1186/s12939-020-01326-w 33302978 PMC7726895

[33] Shi X Zhu D Man X Wang W Zhu K Nicholas S The biggest reform to China’s health system": did the zero-markup drug policy achieve its goal at traditional Chinese medicines county hospitals? Health Policy Plan. (2019) 34(7):483–91. 10.1093/heapol/czz053 31363744

[34] China TCPsGotPsRo. Four departments on the implementation of the pilot reform of the public hospital pay system guidance (2017). Available at: http://www.gov.cn/xinwen/2017-02/10/content_5167037.htm (Cited October 2, 2017).

[35] Jia Q Qian J Shen Y. Research on application status of the national specification for medical service prices. Chin J Health Inform Manage. (2019) 16(02):163–6. 10.3969/j.issn.1672-5166.2019.02.09

[36] Gao L Shi L Meng Q Kong X Guo M Lu F. Effect of healthcare system reforms on public Hospitals’ revenue structures: evidence from Beijing, China. Soc Sci Med. (2021) 283:114210. 10.1016/j.socscimed.2021.114210 34274783

[37] Wang J Li P Wen J. Impacts of the zero mark-up drug policy on hospitalization expenses of copd inpatients in sichuan province, western China: an interrupted time series analysis. BMC Health Serv Res. (2020) 20(1):519. 10.1186/s12913-020-05378-0 32513170 PMC7282107

[38] Peng Z Zhan C Ma X Yao H Chen X Sha X Did the universal zero-markup drug policy lower healthcare expenditures? evidence from Changde, China. BMC Health Serv Res. (2021) 21(1):1205. 10.1186/s12913-021-07211-8 34742310 PMC8571884

[39] Wu D Lam TP Lam KF Zhou XD Sun KS. Health reforms in China: the public’s choices for first-contact care in urban areas. Fam Pract. (2017) 34(2):194–200. 10.1093/fampra/cmw133 28122845

